# The Use of Subcutaneous Injections of Artificial Serum in Eclamptic Albuminura

**Published:** 1897-07

**Authors:** 


### THE USE OF SUBCUTANEOUS INJECTIONS OF ARTIFICIAL SERUM IN ECLAMPTIC ALBUMINURIA.

In *La Presse Medicale Belge* of January 24, 1897, Sole contributes a paper on this subject. He believes that massive injections of artificial serum should be given subcutaneously in all severe cases of toxema. This method was first employed, he states, in 1855, when intravenous injections of salt water were given to combat cholera, and in 1873 the subcutaneous method was employed by Kartz in place of the intravenous procedure. In 1889 Dastre and Loye presented a communication to the the Societe de Biologie in Paris as to the effects of saline injections upon animals, and in 1890 Sahli of Berne attained great success in the treatment of a case of uremia by such injections; in 1893 Porak began using subcutaneous injections in eclampsia to diminish the toxicity of the blood-serum.

Charpentier at the Gynecological Congress in Geneva also recommended this treatment. Later Lejars and Tuffier obtained excellent results by similar methods, but insist that the kidneys must be intact if this method is to be of value. Most of these authors have been satisfied with injecting about half a pint a day.

The following case is interesting as an illustration of the use of this method: On December 7th, a married woman, aged twenty-six years, and a primipara pregnant seven months, was attacked by severe pains in the epigastrium accompanied by nausea and vomiting, occasional headache, and no visual disturbance. She was somewhat stupid, semi-comatose, and she responded with difficulty to questions put to her. There was edema of the lower extremities, of the abdomen, and of the face, which had been present for about five days. The respirations were rapid and shallow, 40 per minute; the pulse small, rapid, filiform, and 160; the temperature was raised. The urine was scanty and contained blood. Rapid analysis showed that it was loaded with albumen, and a more careful examination by means of Esbach's albuminometer showed that there present three drachms of albumen to the quart. One and a half ounces of sulphate of magnesium and some bromide of potassium and chloral were administered by the mouth, and chloroform was given to the point of slight anesthesia. Professor Kuferath was called in consultation, and by the evening the patient was found to be in deep coma and remained so three days later. An application of six leeches was made to the mastoid, and the subcutaneous injection of a small quantity of pilocarpine was resorted to. Later on two severe convulsions involving chiefly the face and upper extremities appeared, and inhalations of chloroform with one injection of pilocarpine were resorted to. Forty minutes later a third and general attack of eclampsia resulted, and chloroform was given again. Three-quarters of an hour later a fourth attack came on. This decided the physician in attendance to bring about artificial delivery, and the patient was given twenty grains of chloral and twenty drops of laudanum by the rectum. Shortly after this the patient began having attacks every half hour, but after a few paroxysms they gradually decreased, the chloral and chloroform being pushed. Twenty-four hours after the onset of the symptoms, manual dilatation of the os was performed and a female child delivered; but two hours later the coma still persisted, respiration was stertorous, and the albuminuria was still present in excess; there was a febrile temperature, and the pulse was rapid. It was then decided to give four ounces of milk with fifteen minims of ether by the rectum, and this was given on two occasions at hour intervals. Later on one ounce of cognac was also added to this injection and repeated twice. The patient's condition, however, did not improve. On the third day after the attack commenced one pint of normal saline solution was injected into the subcutaneous tissues into the axilla of each side, and shortly afterwards catheterization removed from the bladder almost a pint of yellow urine, although the patient in the preceding twenty-four hours had only passed less than half this quantity. On the following day half a pint of fluid was injected into the left flank, and the urine on being measured amounted to nearly a pint. Later on in the same day another injection of half a pint in the right flank was given, and these injections then followed in half-pint quantities every three or four hours, with the result that at the end of twenty-four hours the patient's condition was much ameliorated; the temperature was not so high, the pulse much better, and the respiration nearer the normal. At this time it was noticed that there was a slight eruption upon the skin of the chest, which the author thinks was due to the elimination of the chloride of sodium. The patient ultimately recovered.

The difficulty in most of these cases seems to be that sufficiently large injections are not given. One or two quarts can nearly always be given in twenty-four hours with advantage. The liquid should not flow into the tissues too rapidly, for on the one hand absorption would not be sufficiently active, and on the other the kidneys could not eliminate it.

Each injection should be given in a different place from its predecessors. The advantage of this method of administering saline fluid is that it has no difficulties or dangers, that moderate antisepsis only is required, that a vein does not have to be opened, and that there is no danger of air or other forms of embolism taking place.

The suggestion of Bozzolo that the fluid be injected into the pleural or abdominal cavity is even more dangerous than venous injection; furthermore rectal injection gives very slow results and depends largely upon the ability of the bowel to retain the fluid. Sole believes that these injections should be employed not only in puerperal hemorrhage but also in puerperal septicemia, and mentions four cases of the latter condition which were saved by this method. He quotes one or two authorities, however, as stating that acute inflammation of the kidneys is a contra-indication to this manner of treatment.—*Therapeutic Gazette.*

